# When Melodies Cue Memories: Electrophysiological Correlates of Autobiographically Salient Music Listening in Older Adults

**DOI:** 10.1007/s11357-025-01860-x

**Published:** 2025-10-07

**Authors:** Veronica Vuong, Mary J. O’Neil, Andrew Dimitrijevic, Bradley R. Buchsbaum, Michael H. Thaut, Claude Alain

**Affiliations:** 1 Rotman Research Institute, Baycrest Academy for Research and Education, Toronto, ON Canada; 2 Institute of Medical Science, Temerty Faculty of Medicine, University of Toronto, Toronto, ON Canada; 3 Music and Health Science Research Collaboratory, Faculty of Music, University of Toronto, Toronto, ON Canada; 4 Department of Psychology, Faculty of Health, York University, Toronto, ON Canada; 5 Department of Otolaryngology, Head and Neck Surgery, Sunnybrook Health Sciences Centre, Toronto, ON Canada; 6 Department of Otolaryngology, Head and Neck Surgery, Temerty Faculty of Medicine, University of Toronto, Toronto, ON Canada; 7 Evaluative Clinical Sciences Platform, Sunnybrook Research Institute, Toronto, ON Canada; 8 Department of Physiology, University of Toronto, Toronto, ON Canada; 9 Department of Psychology, University of Toronto, Toronto, ON Canada; 10 Rehabilitation Sciences Institute, Temerty Faculty of Medicine, University of Toronto, Toronto, ON Canada

**Keywords:** Music, Event-related potential, Time-Frequency, Autobiographical memory, Familiarity, Recollection

## Abstract

Autobiographical memory is essential for older adults, providing a foundation for self-identity. Although healthy aging is accompanied by changes in memory retrieval, musical memory remains relatively unaffected, suggesting music may serve as a cue for autobiographical memory recall. Autobiographically salient (ABS) music (i.e., deeply encoded songs associated with significant people, places, and events) is posited to engage distinct memory processes from familiar (FAM) music (i.e., songs that are recognized but lack personal significance). We tested this in 36 older adults (70.6 $\pm$ 6.6 years, 20 females) who listened to music varying by personal significance, including ABS, FAM, and unfamiliar (UFAM) music. In Experiment 1, participants pressed a button as soon as they identified the excerpt as ABS, FAM, or UFAM. In Experiment 2, we measured event-related potentials and time-frequency responses while participants listened to the same stimuli and rated familiarity and memory following each excerpt. Reaction times were fastest for ABS, followed by FAM, then UFAM music. We observed a sustained evoked response from 2238 to 5000 ms post-stimulus onset that was least negative in amplitude for ABS, relative to FAM and UFAM music, over right frontal-central regions. We also observed less beta power suppression for ABS than FAM music between 1300 and 5000 ms over bilateral frontal-central-parietal areas. Our behavioral and neurophysiological findings show that ABS music elicits faster and distinct memory-related neural activity throughout the excerpts compared to FAM music. These results emphasize that music is a powerful cue for activating memory processes, which varies by personal significance.

**Supplementary Information:**

The online version contains supplementary material available at 10.1007/s11357-025-01860-x.

## Introduction

Healthy aging is accompanied by well-documented changes in episodic autobiographical memory retrieval. Compared to young adults, older adults tend to recall episodic memories with fewer event-related details [1, 2], less specificity [2, 3] and diminished vividness [4]. Autobiographical memory is organized in a hierarchical model consisting of three levels with increasing specificity (see Conway and Pleydell-Pearce [5]). Successful retrieval can occur as a cascading effect, whereby activating higher-level representations facilitates access to event-specific details, leading to mental time travel [6]. Crucially, this process is influenced by the saliency of the cue.

Music can be a highly effective stimulus to trigger autobiographical memories [7]. For example, compared to words [8] and faces [9], music has been shown to elicit more episodic details. While the effectiveness of music as a memory cue may be attributed to the frequency with which it is listened to in daily life [10], the emotional quality it possesses [11], and/or associations to significant events [12], not all music is encoded into memory to the same degree. For instance, unfamiliar (UFAM) music refers to novel songs that we have not encountered before and, thus, neither recognize nor regard as personally significant. Familiar (FAM) music includes those we recognize through exposure but lack personal meaning. Autobiographically salient (ABS) music is deeply encoded and is distinguished by its personal significance, evoking specific memories (i.e., a person, location, or event) and associated emotions. Remarkably, the capacity for ABS music to cue autobiographical memories persists in individuals with mild cognitive impairment and Alzheimer’s disease [13–16], underscoring music’s role as a bridge to the past. Understanding how the brain differentially responds to music varying in memory-related significance is pivotal to developing and implementing effective music-based interventions for older adults, particularly those with dementia.

Given the distinct characteristics of ABS, FAM, and UFAM music, each type is expected to elicit different patterns of neural activity, influenced by their varying degrees of personal significance. Previous neuroimaging research using functional magnetic resonance imaging (fMRI) and positron emission tomography (PET) has primarily focused on FAM and UFAM music [17]. Our Activation Likelihood Estimation meta-analysis of 23 neuroimaging studies showed that listening to FAM compared to UFAM music, or an equivalent control condition, yielded consistent activity in the left supplementary motor areas, left inferior frontal gyrus, and the left claustrum/insula [17]. Fewer studies have examined ABS relative to UFAM music; however, greater activation has been reported for ABS than UFAM music in the medial prefrontal cortex, precuneus, anterior insula, basal ganglia, hippocampus, amygdala, and cerebellum [18]. Given the above findings, different neural substrates may support ABS and FAM music. However, research that directly compares the two music conditions is lacking.

The temporal aspects underlying the feeling of familiarity may also vary. Prior studies used a gating paradigm where successive musical notes [19] or the duration of the excerpt [20–22] were gradually increased to assess the minimum amount of acoustic information (e.g., timbre or pitch) required to evoke a feeling of familiarity. Findings from these studies suggest that 200 to 500 ms in stimulus duration [20–22] is needed to elicit a sense of familiarity but is insufficient to yield above-chance level identification of popular songs [23]. While a feeling of familiarity may quickly emerge from hearing a musical excerpt, it is unclear how long listeners need to recognize excerpts associated with their autobiographical memory. Listeners may recognize ABS music more quickly due to deeper encoding. Moreover, the duration for familiarity and recognition discussed above are based on young adult studies and may be longer in older adults due to age-related changes in sensory and cognitive processing.

Scalp-recordings of event-related potentials (ERPs) provide precise temporal information and can differentiate neural activity underlying familiarity and recollection during episodic memory retrieval [24]. For instance, correctly identified old items elicit more positive-going waveforms than new items. In studies examining familiarity, this old/new effect has been observed over the mid-frontal scalp area, referred to as the FN400 (for a review, see Friedman and Johnson [25]). Recollection is indexed by an old/new effect over the left parietal scalp region between 400 and 800 ms post-stimulus onset, termed the Late Positive Component (LPC) [26]. A third relevant ERP modulation is a right-lateralized Late Frontal Effect (LFE), emerging 600 to 2000 ms after stimulus onset [27]. The LFE is thought to index post-retrieval processing of contextual information [27, 28] or self-relevant processing [29, 30]. While these neural correlates have been identified using study-test paradigms that comprised of words [31] or faces [32, 33], similar ERP modulations could also underlie episodic retrieval during music listening.

### Music as a memory cue

In recent years, there has been growing interest in understanding how music may engage episodic memory. For instance, Jagiello et al. [34] presented young adults with an ABS song selected by the participant and compared this with an experimenter-chosen UFAM excerpt. They reported an early ERP modulation from 350 to 750 ms post-stimulus onset over right frontal-temporal regions, which exhibited greater negativity for ABS than UFAM music. They also observed a subsequent ERP modulation, from 540 to 750 ms after music onset over the left parietal area, characterized by an increase in amplitude for ABS relative to UFAM music. A more recent study observed greater positivity for FAM than UFAM music from 400 to 450 ms over right and left frontal-central scalp regions [35]. This was accompanied by greater alpha power suppression when listening to FAM than UFAM music, over left frontal and posterior areas, along with low beta suppression over frontal scalp areas. The former is interpreted as increased attentional engagement, while the latter, as familiarity-related processes. Although familiarity ratings were obtained, the possibility that some of the excerpts may have elicited an autobiographical memory cannot be excluded. The above studies shed light on the neural correlates of musical memory but were limited to only two experimental conditions. Thus, it remains unclear whether ABS music generates different neural responses than FAM music. A comparison of the neural responses to ABS, FAM, and UFAM music could provide deeper insight into the neural underpinnings of music perception and memory.

The present study used behavioral and electroencephalography (EEG) to examine how healthy older adults process ABS, FAM, and UFAM music. In Experiment 1, we measured the time needed by older adults to recognize ABS, FAM, and UFAM music by asking participants to press a button as soon as they identified the excerpt as belonging to the respective music condition. Experiment 2 investigated ERPs and time-frequency responses across music conditions in the same group of participants. Familiarity and memory ratings were provided at the end of each excerpt to minimize the contamination of motor processes during music listening. We anticipated that the ERPs of the music conditions would differ in their sustained activity, demonstrating distinct memory processes associated with each contrast. Specifically, we hypothesized that comparing FAM and UFAM music would reveal an early response indexing familiarity. ABS music relative to UFAM music would also produce an early response indexing familiarity and subsequent difference in neural activity that may reflect a greater strength of memory association. Lastly, ABS music compared to FAM music would reveal an LFE, indexing greater self-relevant processing or post-retrieval elaboration.

## Methods

### Participants

Participants were recruited if they were generally healthy, 60 years of age or older, fluent in English, had normal or corrected-to-normal vision and hearing, were non-musicians (i.e., individuals without a bachelor’s degree in music or equivalent, such as Royal Conservatory of Music [RCM] training at the Associate of the Royal Conservatory of Music [ARCT] level), attained a minimum of high school education, reported no diagnoses and/or hospitalizations due to head injury, depression, anxiety, psychiatric disorder, seizure disorder, alcohol or substance abuse, learning disabilities, tinnitus, major medical events/surgeries (e.g., open heart surgery), and brain radiation. All participants were screened to exclude medications affecting cognitive function and brain activity.

Previous ERP studies showed differences between FAM and UFAM music [35] and ABS and UFAM music [34], but used relatively small sample sizes (*n* = 20 and *n* = 10, respectively) that were limited to young adults. We recruited a larger sample, due to the higher variability of older adults’ behavioral and brain responses [36, 37]. Data were excluded from two participants due to non-compliance with the task(s), and two additional participants were excluded because of noisy electrophysiological data (e.g., excessive ocular and muscle artifacts). As a result, the final sample included 36 older adults (range = 61-86 years of age, 20 females. See Table 1 for demographic characteristics). All participants completed Experiment 1 and Experiment 2 on the same day.

**Table 1:** Demographic Characteristics (*n* = 36)

Variable	Mean (*SD*)
Age (Years)	70.6 (6.6)
Sex at Birth (F:M)	20:16
Handedness (R:L)	34:2
Bilateral Pure Tone Average Threshold (db HL)	19.4 (9.6)
Montreal Cognitive Assessment	28 (1.6)
Amount of Time Playing Instrument(s) (Years)	4.2 (10.9)

*F* = Female; *M* = Male; *R* = Right; *L* = Left; *db HL* = Decibels Hearing Level; *SD* = Standard Deviation.

This study was approved by the Research Ethics Board at the Rotman Research Institute, Baycrest Academy for Research and Education. Participants were recruited from the Rotman Research Institute participant database. All participants provided informed consent and received monetary compensation ($15/hour) plus transportation costs.

### Pre-experiment interview

Prior to the experimental sessions, we conducted an interview to identify 15 ABS musical titles with artist name(s) per participant, emphasizing that ABS music must bear personal significance (i.e., prompts a memory of a person(s), location, or event). Since popular music, such as radio hits, typically contains vocals, and taking into consideration neural differences that may occur from listening to vocal vs. instrumental music, it was specified that all ABS songs must contain English vocals.

Using each ABS song, a FAM and UFAM song was selected and matched, to the best of our abilities, on the following aspects:

1. Artist style/genre.
2. Released the same or around the same year (± 5 years).
3. Number of online streams/plays: While we acknowledge that purchasing music albums was more commonly practiced among older adults, consistent data on album sales are not readily accessible. Currently, online streaming is the dominant mode of music consumption. According to the Recording Industry Association of America [38], a song is certified Gold once it reaches 500,000 units (1 certification unit = 1 paid download or 150 streams), equating to 75 million plays across platforms. Spotify and YouTube are presently two of the largest streaming mediums and additionally provide play counts. We used a combined value of 50 million plays as a metric for FAM songs. Although this threshold is lower than the 75 million plays required for Gold certification, it does not include paid downloads or streams from other platforms such as Apple Music, Amazon Music, Tidal, Deezer, Pandora, and Bandcamp. All UFAM songs had fewer than 3 million combined plays on Spotify and YouTube. We selected the highest indicated number of streams in the event of multiple YouTube videos.

We utilized Spotify’s recommendation algorithm, which suggests songs with similar attributes. We refrained from selecting FAM and UFAM matches by the same ABS artist(s). All selected songs were reviewed by a musician on the study team to ensure that the songs matched appropriately. Although we did not initially choose FAM songs based on whether they were on the Billboard charts, we recognize that this metric might be more relevant for older songs (e.g., from the 1940s or 1950s), which precede the development of streaming platforms. We subsequently collected this data for each song (see Table 1 in the Supplementary Material for examples).

## Experiment 1: Behavioral Study

### Materials & Methods

#### Stimuli

Stimuli were music excerpts from the participant’s playlist (i.e., ABS music) and matched FAM and UFAM music. Each excerpt was trimmed to 10 seconds at a salient part of the song (e.g., the chorus) using Audacity® (version 3.2.2) [39]. Then, using a custom script in Praat (version 6.4.05) [40], the mean intensity was scaled to 70 decibels (dB) sound pressure level (SPL). An acoustic analysis (not shown) revealed that the excerpts were broadly comparable across conditions on properties including root mean square intensity, tempo, and zero crossing rate. While significant differences were observed in onset rise time, pitch, spectral centroid and spectral bandwidth, these were relatively modest in magnitude.

### Procedure

First, participants familiarized themselves with the task by completing four practice trials with music excerpts not used in the experimental run. Then, participants completed five blocks of 45 trials each (15 trials per music type). Thus, each excerpt was presented five times. Within each block, the excerpts from the ABS, FAM, and UFAM conditions were presented randomly. Music excerpts were presented binaurally via insert earphones at 55 dB SPL.

On each trial, participants heard an excerpt from one of three conditions. They indicated whether it belonged to the ABS, FAM, or UFAM category as quickly and accurately as possible by pressing one of three keys on a computer keyboard. ABS music was specified as personally significant; FAM music was indicated as music that was recognized but lacked personal significance; and UFAM music was defined as music that had not been heard before. We emphasized that these categorizations of music were to be framed within their lifetime (i.e., hearing a UFAM clip for the second time does not qualify as a familiar stimulus and, therefore, would still be considered as belonging to the UFAM condition). The music stopped once the key was pressed, and the subsequent trial began three seconds later. The button assignment was counterbalanced across participants, resulting in six possible configurations. No feedback was provided after each trial or block. The experiment was programmed using Presentation software (version 23.0) [41].

### Statistical analysis

#### Behavioral analysis

To verify that the selected stimuli were appropriately categorized based on familiarity and memory-related processing, we examined participants’ classification accuracy and reaction times (RT) to ABS, FAM, and UFAM excerpts. A repeated-measures ANOVA was performed separately for classification accuracy and RT from correct responses, with music condition as the within-subjects factor, using R software [42]. Sphericity was tested using Mauchly’s test (*W*). When applicable, the degrees of freedom were adjusted using Greenhouse-Geiser (*ε*) estimates. Bonferroni corrections were applied for pairwise comparisons and are reported herein.

## Results

### Behavioral results

**Fig. 1:**
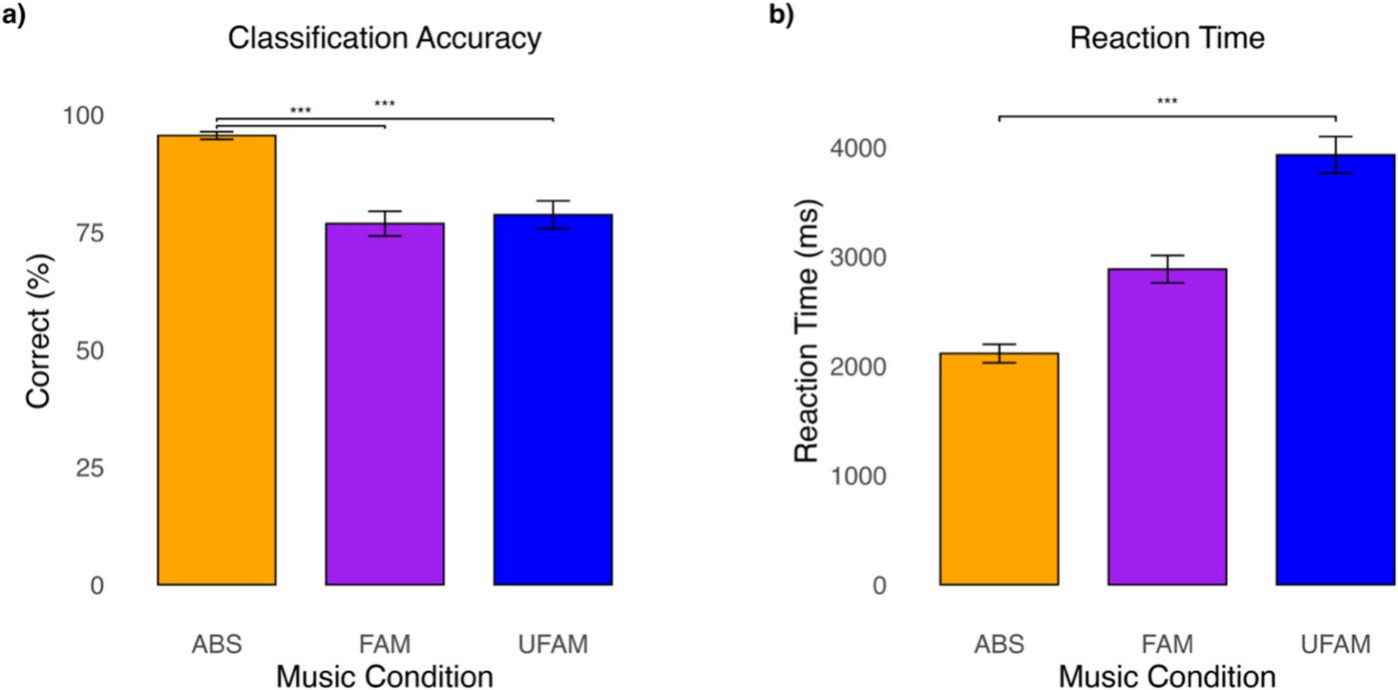
Bar graphs from the music listening task in Experiment 1, depicting **a**) the percentage of correct responses when classifying music excerpts by music condition and **b**) mean reaction time based on correct responses. Error bars indicate the standard error of the mean (SEM). Classification accuracy was highest for ABS music (orange), followed by UFAM music (blue), then FAM music (purple). Reaction time was fastest for ABS music, followed by FAM music, then UFAM music. *ABS* = Autobiographically salient; *FAM* = Familiar; *UFAM* = Unfamiliar, *** *p* < 0.001

Figure 1a and b show the group average classification accuracy and RT for each music condition. The repeated-measures ANOVA on classification accuracy yielded a main effect of condition (*F*_(2,70)_ = 24.90, *p* < 0.001, $\eta$ ^2^_p_ = 0.27). Pairwise comparisons revealed significantly higher correct responses when classifying ABS music (*M* = 95.5%) than FAM (*M* = 76.8%) and UFAM (*M* = 78.7%) music (*p* < 0.001 in both cases). Although no significant difference emerged between FAM and UFAM music (*p* = 1), classification accuracy remained above chance, indicating that participants could differentiate among conditions. The percentage of trials in which ABS music was misidentified as FAM and UFAM music was 3.6% and 0.8%, respectively. Participants incorrectly identified FAM music as ABS and UFAM music in 10.0% and 13.1% of trials. Lastly, in trials involving UFAM music, 20.0% and 1.3% of trials were incorrectly identified as FAM and ABS music, respectively.

The repeated-measures ANOVA also revealed a main effect of condition on RT (*F*_(2,70)_ = 92.95, *p* < 0.001, $\eta$ ^2^_p_ = 0.48). ABS music was identified the fastest (*M* = 2114 ms, *SD* = 506 ms), intermediate for FAM music (*M* = 2885 ms, *SD* = 754 ms), and slowest for UFAM music (*M* = 3930 ms, *SD* = 1003 ms). Pairwise comparisons showed that all music conditions significantly differed from each other (*p* < 0.001 for all comparisons).

## Experiment 2: EEG Study

### Materials & methods

#### Stimuli

For each participant, the same set of music excerpts from Experiment 1 was used, with the only difference being that they were shortened to five seconds using Audacity (version 3.2.2) [39]. This duration was motivated by the findings from Experiment 1 and allowed us to maintain the same number of trials within the time constraints of the EEG session.

### Procedure

Participants were instructed to avoid movement as much as possible during EEG recording. First, two practice trials with music excerpts not used in the experimental run were presented. Then, participants completed five blocks of 45 trials each, in which the ABS, FAM, and UFAM excerpts were presented in a random order, with an inter-trial interval of three seconds. The music excerpts were presented binaurally through insert earphones at the same intensity as in Experiment 1. During music listening, participants were asked to direct their gaze to a white fixation cross in the center of a black screen. After each music excerpt, participants used a 4-point scale to rate familiarity and strength of memory association (1 = No familiarity/memory association; 2 = Some familiarity/memory association; 3 = Moderate familiarity/memory association; 4 = High familiarity/memory association) using the keyboard. The ratings were self-paced. The next trial was initiated once the memory rating was completed.

### Electroencephalography acquisition and processing

Neuroelectric brain activity was recorded continuously using a 64-channel BioSemi ActiveTwo acquisition system and digitized at a sampling rate of 2048 Hz with a bandpass of DC-100 Hz. The electrodes were positioned on the scalp according to the standard 10-20 system. A Common Mode Sense active and a Driven Right Leg passive electrode, placed near the P1 and P2 electrodes, form the ground loop for common mode rejection. Voltage offsets were kept below ± 20 mV.

EEG data were processed offline using Brain Electrical Source Analysis (BESA) Research software (version 7.1). Defective electrodes were interpolated using values from surrounding electrodes. Up to seven electrodes were interpolated per participant. Artifacts from ocular movements were identified from the recording and used to generate spatial components that model ocular artifacts, such as eye blinks as well as vertical and lateral eye movements. The spatial components were then subtracted from the continuous EEG to correct for eye movements and eye blinks. The data were downsampled to 256 Hz, re-referenced to the average of all electrodes, and digitally filtered with a 0.01 Hz high-pass filter (forward phase, 6 dB/octave) and a 30 Hz low-pass filter (zero phase, 24 dB/octave). Data were segmented into epochs, including 500 ms pre-stimulus and 6000 ms post-stimulus activity. The EEG recordings were scanned for artifacts. Trials contaminated by excessive peak-to-peak deflection (120 µV) were marked and excluded from the analysis. On average, 95.1% of trials were accepted for each music condition. The analysis epochs were averaged and baseline-corrected by subtracting the mean voltage within the pre-stimulus interval (−500 to 0 ms), from each time point, applied independently for each electrode.

### Time-frequency analysis

The continuous EEG data were decomposed into time-frequency representation using complex demodulation with a 100-ms time window and 0.5 Hz frequency resolution from 1 to 30 Hz. We defined the frequency ranges as follows: Delta (1 to 4 Hz), theta (4 to 8 Hz), alpha (8 to 12 Hz), low beta (12 to 16 Hz), mid beta (16 to 20 Hz), and high beta (20 to 30 Hz). Baseline correction was applied using a pre-stimulus interval from −500 to 0 ms.

### Statistical analyses

#### Behavioral data analysis

A one-way repeated-measures ANOVA was performed separately to assess the effects of music conditions on familiarity and memory ratings. A Greenhouse-Geisser correction was used when the assumption of sphericity was violated. Planned pairwise comparisons were conducted using Bonferroni correction to examine significant main effects. Statistical analyses were performed using R software [42].

### ERP and time-frequency analyses

ERP waveforms and time-frequency representations were analyzed using a within-group ANOVA in BESA Statistics software (version 2.1). This software identifies clusters in time and space and uses a series of F-tests to compare the amplitude between experimental music conditions at every time point. Preliminary clusters were identified in time (adjacent time points) and space (adjacent electrodes) where brain responses differed between the experimental conditions. The neighbor distance (circle arc length between two points) was set to 4 cm and the significance level of cluster building was set to 0.05, with 5000 permutations. We examined brain responses (ERPs and time-frequency representations) across conditions from 0 to 5000 ms. Only significant clusters with at least four channels were included.

### Brain-behavior correlation analyses

Correlation analyses were computed between participants’ neuroelectric measures, including ERP amplitude, time-frequency responses and behavioral measures, including RT, familiarity, and memory ratings. Analyses were conducted separately within each music condition. Using BESA Statistics (version 2.1), Pearson correlation coefficients were calculated across participants for each electrode and time point (for ERP data), as well as frequency bin (for time-frequency data), generating spatiotemporal correlation maps. A time window of 0 to 5000 ms was specified. Significant correlation clusters were identified using a cluster-based permutation approach with Monte-Carlo resampling [43], (*p <* 0.05, 5000 permutations), with correction for multiple comparisons across electrodes and time/time-frequency. Similar to the ERP and time-frequency analyses, only significant clusters with at least four channels were included.

## Results

### Behavioral data results

**Fig. 2:**
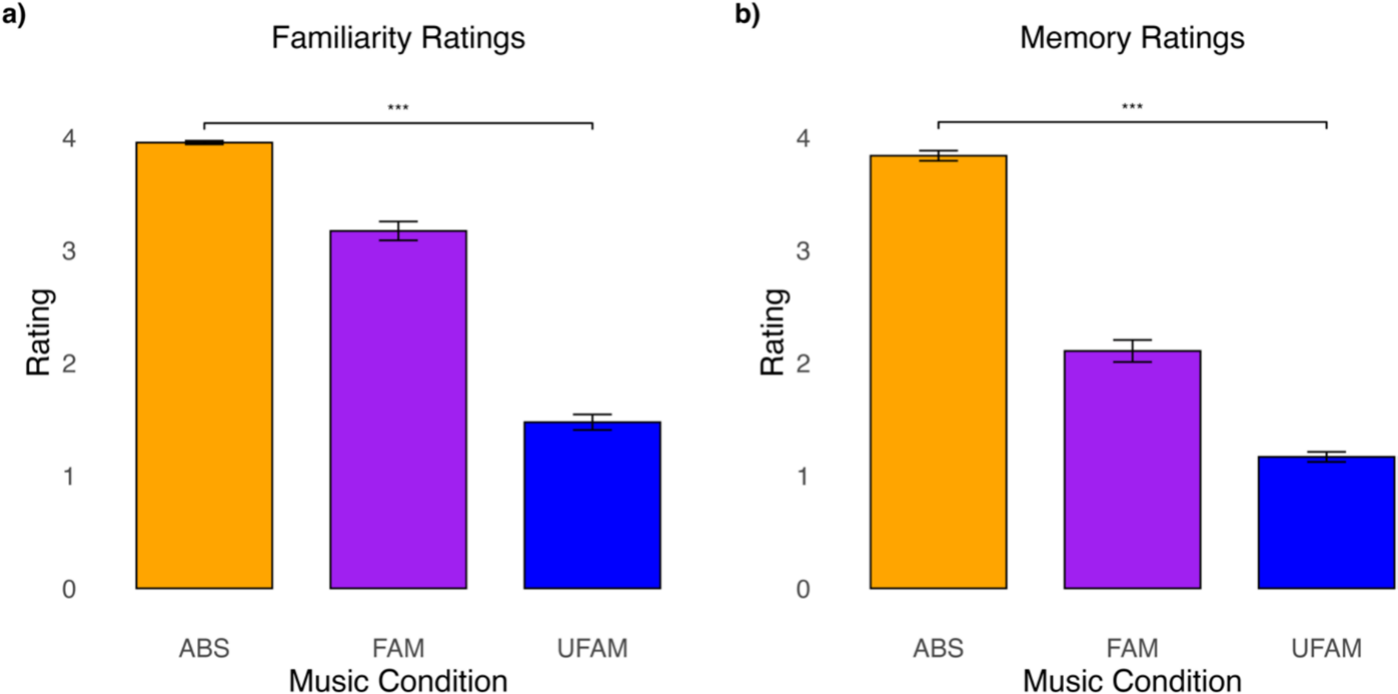
Bar graphs from the music listening task in Experiment 2, depicting **a**) mean familiarity and **b**) mean memory ratings on a 4-point scale, by music condition. Error bars indicate the standard error of the mean (SEM). Familiarity and memory ratings were highest for ABS music (orange), followed by FAM (purple), then UFAM music (blue). *ABS* = Autobiographically salient; *FAM* = Familiar; *UFAM* = Unfamiliar, *** *p* < 0.001

Figure 2a and b show the group mean familiarity and memory rating as a function of music condition. The one-way repeated-measures ANOVA yielded a main effect of condition on mean familiarity ratings (*F*_(2,70)_ = 467.18, *p* < 0.001, $\eta$ ^2^_p_ = 0.881). Familiarity ratings were highest for ABS music (*M* = 3.95, *SD* = 0.09), followed by FAM music (*M* = 3.17, *SD* = 0.51), and lowest for UFAM music (*M* = 1.47, *SD* = 0.41) (all pairwise comparisons *p* < 0.001).

A one-way repeated-measures ANOVA on memory ratings also showed a main effect of condition (*F*_(2,70)_ = 533.36, *p* < 0.001, $\eta$ ^2^_p_ = 0.883), with the highest memory ratings for ABS music (*M* = 3.83, *SD* = 0.27), then FAM music (*M* = 2.10, *SD* = 0.59), and lastly, UFAM music (*M* = 1.16, *SD* = 0.26) (Fig. 2b) (all pairwise comparisons *p* < 0.001).

### ERP results

**Fig. 3:**
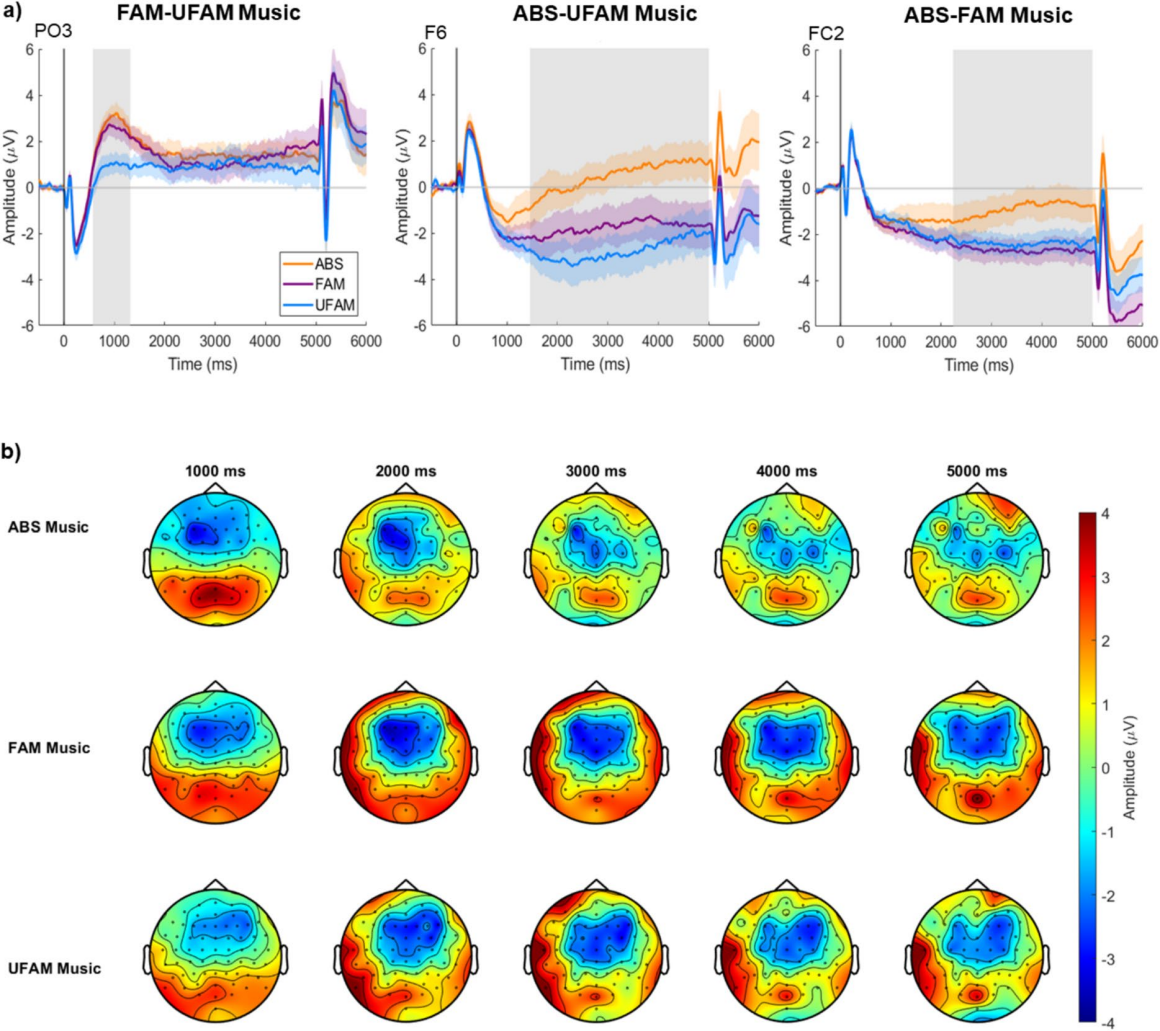
**a**) Grand-averaged waveforms recorded at left parietal-occipital (PO3), right frontal (F6) and right frontal-central (FC2) electrodes, elicited by ABS music (orange), FAM music (purple), and UFAM music (blue), time-locked to the onset of music stimuli. Shading around each line represents the mean ± standard error of the mean (SEM). The gray box represents the time window where event-related potential (ERP) amplitudes significantly differed between planned pairwise contrasts. Positive voltage is plotted upwards. *ABS* = Autobiographically salient; *FAM* = Familiar; *UFAM* = Unfamiliar. **b**) Topographic maps illustrating the grand-averaged ERP amplitude distributions during music listening, shown for each condition across time from 1000 to 5000 ms. Rows correspond to music conditions: ABS (top row), FAM (middle row), and UFAM (bottom row). Columns represent successive time points. Voltage values reflect the average across participants at each electrode. *ABS* = Autobiographically salient; *FAM* = Familiar; *UFAM* = Unfamiliar

Figure 3a and b show the group mean ERP waveforms and topographies elicited by ABS, FAM, and UFAM music, which were time-locked to the onset of the music excerpt. All music conditions generated transient onset responses that inverted polarity between frontal-central and temporal-parietal electrodes, consistent with neural generators in the planum temporale and Heschl’s gyrus along the superior temporal gyrus [44, 45]. These transient responses were followed by a large positive wave over the parietal region, a sustained evoked potential largest over right frontal-central and offset responses largest over midline central areas.

When examining evoked activity from 0 to 5000 ms, cluster-based statistics revealed two significant clusters for the FAM and UFAM pairwise contrast. The first cluster was observed between 574 and 1316 ms, whereby FAM music was more positive in amplitude than UFAM music over left parietal-occipital electrodes, similar to an LPC. The second cluster, representing a polarity reversal, was observed between 770 and 1637 ms over left frontal-central regions.

The ABS and UFAM pairwise contrast indicated four significant clusters, with the most significant cluster observed between 1457 and 5000 ms (*p* < 0.001) over the right frontal cortex. The waveform for UFAM music displayed greater sustained negativity than ABS music, with the latter condition steadily rising above the zero-voltage line over time.

Lastly, the ABS and FAM pairwise contrast yielded two significant clusters. The first cluster exhibited the most prominent effect, from 2238 to 5000 ms post-stimulus onset (*p* < 0.001), indicating more negative sustained ERP amplitude when listening to FAM than ABS music. This modulation was largest over the right frontal-central region. For a detailed list of all clusters, see Table 2.

**Table 2:** Summary of Cluster-Based Permutation Statistics from ERP Analysis

FAM Music – UFAM Music					
Cluster	Mean FAM	Mean UFAM	*p*-value	Latency (ms), Maximum Latency (ms)	Electrodes
1	2.2019	0.8990	0.0018	574-1316, *645*	P3, P5, *PO3*, O1
2	−2.5031	−1.412	0.003	770-1637, *1078*	F1, FC5, FC3, *FC1*

ABS Music – UFAM Music					
Cluster	Mean ABS	Mean UFAM	*p*-value	Latency (ms), Maximum Latency (ms)	Electrodes
1	−0.4295	−2.6457	0	1457-5000, *1957*	AF8, AF3, Fz, F2, F4, *F6*, FC6, FC4, FC2
2	2.5097	1.2566	0.0004	578-1383, *977*	P1, P3, P5, P7, *PO3*, O1, POz, Pz, CP2, P2, P6, PO4, O2
3	−2.3824	−1.2204	0.0032	602-1344, *816*	F1, F3, FC5, *FC3*, FC1, FCz
4	0.9938	3.2268	0.0202	2809-3719, *3520*	CP5, P7, *P9*, PO7

ABS Music – FAM Music					
Cluster	Mean ABS	Mean FAM	*p*-value	Latency (ms), Maximum Latency (ms)	Electrodes
1	−0.3851	−2.4194	0	2238-5000*, 3727*	F1, FC1, AF8, Fz, F2, F4, F6, FC4, *FC2*, C2
2	−0.2199	1.8752	0.0002	1762-4754, *2645*	FT8, T8, TP8, P8, *P10*

*ABS* = Autobiographically salient; *FAM* = Familiar; ms = Millisecond; *UFAM* = Unfamiliar; *Italicized* = Electrode or latency at maximum amplitude

### Early music-related memory effect

A previous study [35] reported differences between FAM and UFAM excerpts within the first 1000 ms. For comparison, we performed an additional analysis from 0 to 1000 ms to determine if we would observe an FN400 for the FAM-UFAM music contrast using a shorter time window. We found greater negativity for FAM than UFAM music from 589 to 1000 ms over left frontal-central scalp regions (F1, F3, F5, FT7, FC5, FC3, FC1, FCz). The cluster peaked at 816 ms at the FC3 electrode.

### Time-frequency results

**Fig. 4:**
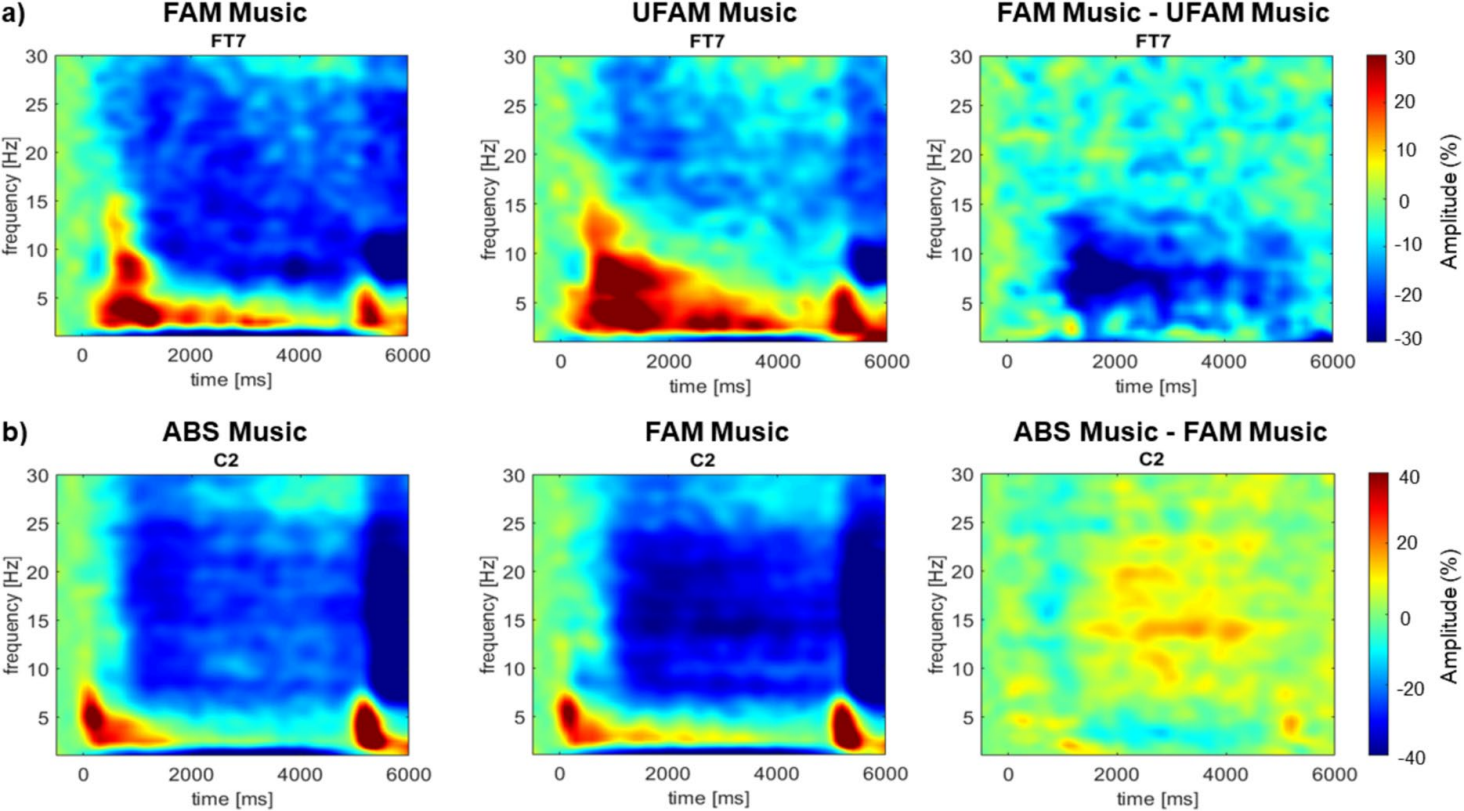
Group mean time-frequency representations over the **a**) left frontal-temporal (FT7) and **b**) midline-right central region (C2). Time 0 indicates stimulus onset. The color scale represents percent power change relative to the baseline. Greater alpha and low beta suppression is observed when listening to FAM compared to UFAM music (top row) and ABS music (bottom row). *ABS* = Autobiographically salient; *FAM* = Familiar; *UFAM* = Unfamiliar

Figure 4 shows the group mean temporal spectral evolution spectrograms for the three conditions and specific contrasts between FAM and UFAM, as well as between ABS and FAM conditions at the peak electrode. For all condition-specific spectrograms, transient theta power can be observed at music onset and offset. In addition, FAM and UFAM music showed alpha and beta synchronization between 500 and 1500 ms after stimulus onset.

When comparing FAM and UFAM music, the cluster analysis procedure and permutation-based statistics revealed one significant spatiotemporal cluster (*p* < 0.001). The cluster started at stimulus onset and ended at 5000 ms, encompassing delta, theta, alpha, and beta bands. It peaked at 1800 ms and was maximum in the alpha band (11 Hz) over the left frontal-temporal scalp area (FT7), revealing greater suppression for FAM music than UFAM music (Fig. 4a).

Two significant clusters were identified for the ABS and UFAM music contrast (not shown). The most significant cluster (*p* < 0.001) spanned the entire epoch and encompassed all frequencies, with a peak in alpha power (11.5 Hz) over the left central scalp area (C1). Greater suppression for ABS music relative to UFAM music was observed. This contrast was outside our interest, thus, the time-frequency response is not shown here.

The ABS and FAM music contrast revealed three significant clusters. The primary significant cluster (*p* < 0.001) began at 1200 ms after stimulus onset and lasted until the end of the music excerpt, extending across theta, alpha, and beta bands. The maximum difference occurred in the beta band (14 Hz) at 3400 ms over the right central scalp area (C2), indicating greater suppression for FAM than ABS music (Fig. 4b).

Previous research showed greater suppression of alpha and low beta power when listening to FAM than UFAM music [35]. Based on these findings, we compared alpha (8 Hz to 12 Hz) and low beta (12 Hz to 16 Hz) bands across conditions.

### Frequency band specific analysis

#### Alpha frequency band

**Fig. 5:**
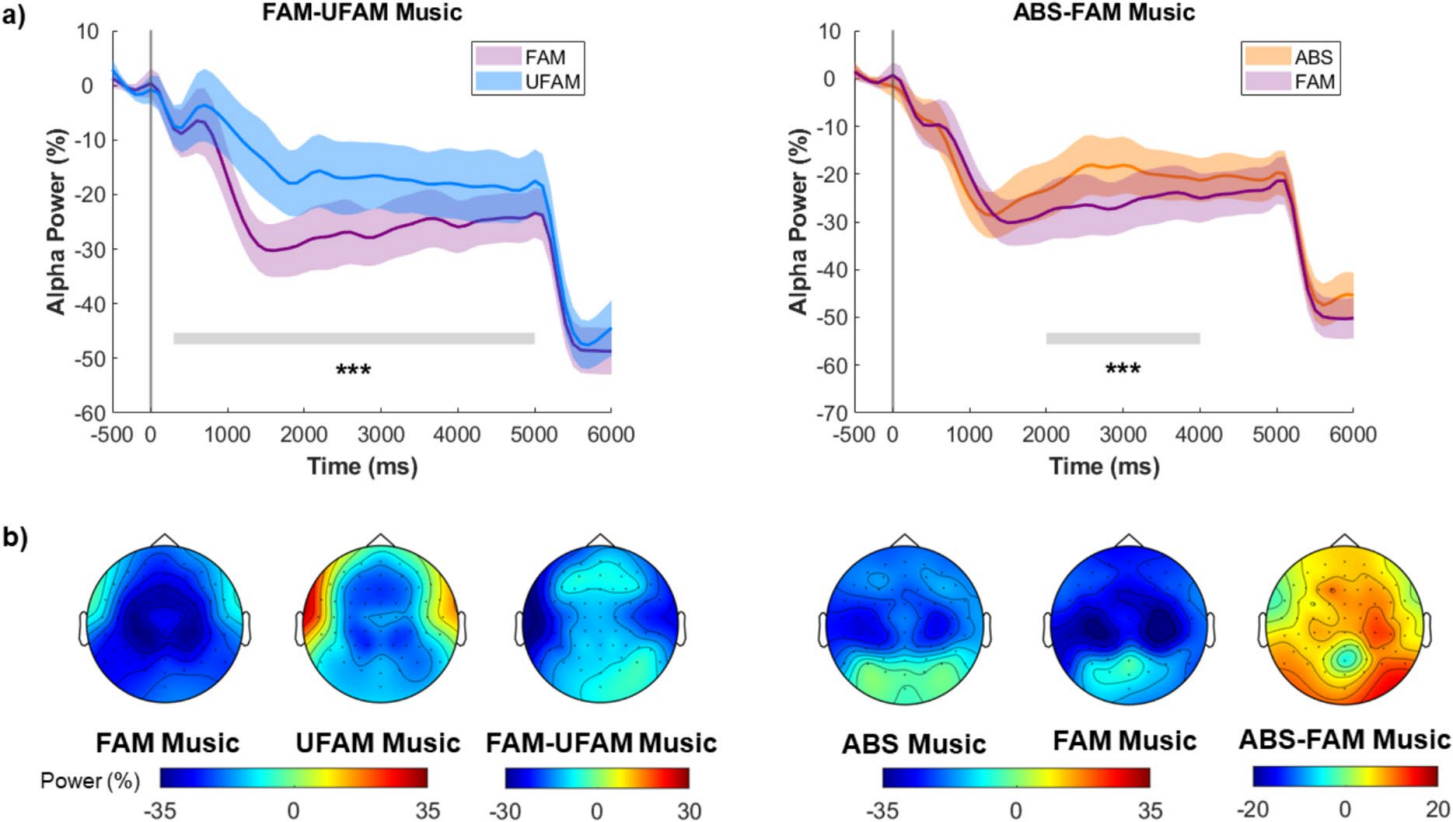
**a**) Time courses of alpha power (8 to 12 Hz) during music listening, shown relative to the pre-stimulus baseline, averaged across participants and electrodes within each significant cluster. Colored lines represent each condition (UFAM = blue, FAM = purple, ABS = orange). Shading around each line represents ±1 SEM. Time 0 indicates the onset of the stimuli. The grey bar denotes the time window of significant differences, *** *p* < 0.001. Greater alpha suppression is observed for FAM music compared to UFAM music (left panel) and ABS music (right panel). *ABS* = Autobiographically salient; *FAM* = Familiar; *UFAM* = Unfamiliar **b**) Topographic maps showing alpha power (8 to 12 Hz) distribution around the peak latency (± 300 ms). The left panel displays the distribution while listening to FAM, UFAM, and FAM-UFAM music from 1000 to 1600 ms (peak = 1300 ms); the right panel displays ABS, FAM, and ABS-FAM music from 2300 to 2900 ms (peak = 2600 ms). Power values reflect percent change from baseline, averaged across participants and displayed at each electrode. The FAM-UFAM difference map shows greater alpha suppression for FAM music, predominantly over bilateral temporal regions, with maximal effects over the left temporal cortex. The ABS-FAM difference map reveals greater suppression for FAM than ABS, with the strongest difference over the right parietal region

Figure 5a shows the temporal dynamics of alpha power activity, averaged across electrodes identified from cluster-based permutation statistics.

When examining changes in alpha power over time, one cluster was observed for the FAM-UFAM music contrast, revealing a reduction in alpha power for the former condition compared to the latter (*p* < 0.001). This cluster was observed between 300 and 5000 ms. The peak occurred at 1300 ms with the strongest difference over the left temporal cortex (Fig. 5b, left panel).

The ABS-FAM music contrast also revealed greater alpha suppression for FAM relative to ABS music (*p* < 0.001). The effect occurred between 2000 and 4000 ms, peaking at 2600 ms and was right-lateralized overparietal areas (Fig. 5b, right panel). For details on the cluster-based permutation statistics, see Table 3.

**Table 3:** Summary of Cluster-Based Permutation Statistics from Alpha Band Power Analysis

FAM Music – UFAM Music					
Cluster	Mean FAM	Mean UFAM	*p*-value	Latency, Maximum Latency (ms)	Electrodes
1	−0.2734	−0.1389	0	300-5000, *1300*	Fp1, AF7, F1, F3, F5, F7, FT7, FC5,
					FC3, FC1, C1, C3, C5, T7, TP7, CP5, CP3, CP1, P1, P3, P5, P7, *P9*, PO7, O1, Pz, CPz, Fpz, Fp2, AF8, AF4, AFz, Fz, F2, F4, F6, F8, FT8, FC6, FC4, FC2, FCz, Cz, C2, C4, C6, T8, TP8, CP6, CP4, CP2,P4

ABS Music – FAM Music					
Cluster	Mean ABS	Mean FAM	*p*-value	Latency, Maximum Latency (ms)	Electrodes
1	−0.2056	−0.3088	0	2000-4000*, 2600*	F1, F3, FC1, AFz, Fz, F2, F4, F6, FC6, FC4, FC2, FCz, Cz, C2, C4, CP6, CP4, CP2, P6, P8, *P10*, PO8

*ABS* = Autobiographically salient; *FAM* = Familiar; *ms* = Millisecond; *UFAM* = Unfamiliar; *Italicized* = Electrode at maximum amplitude

#### Low beta frequency band

**Fig. 6:**
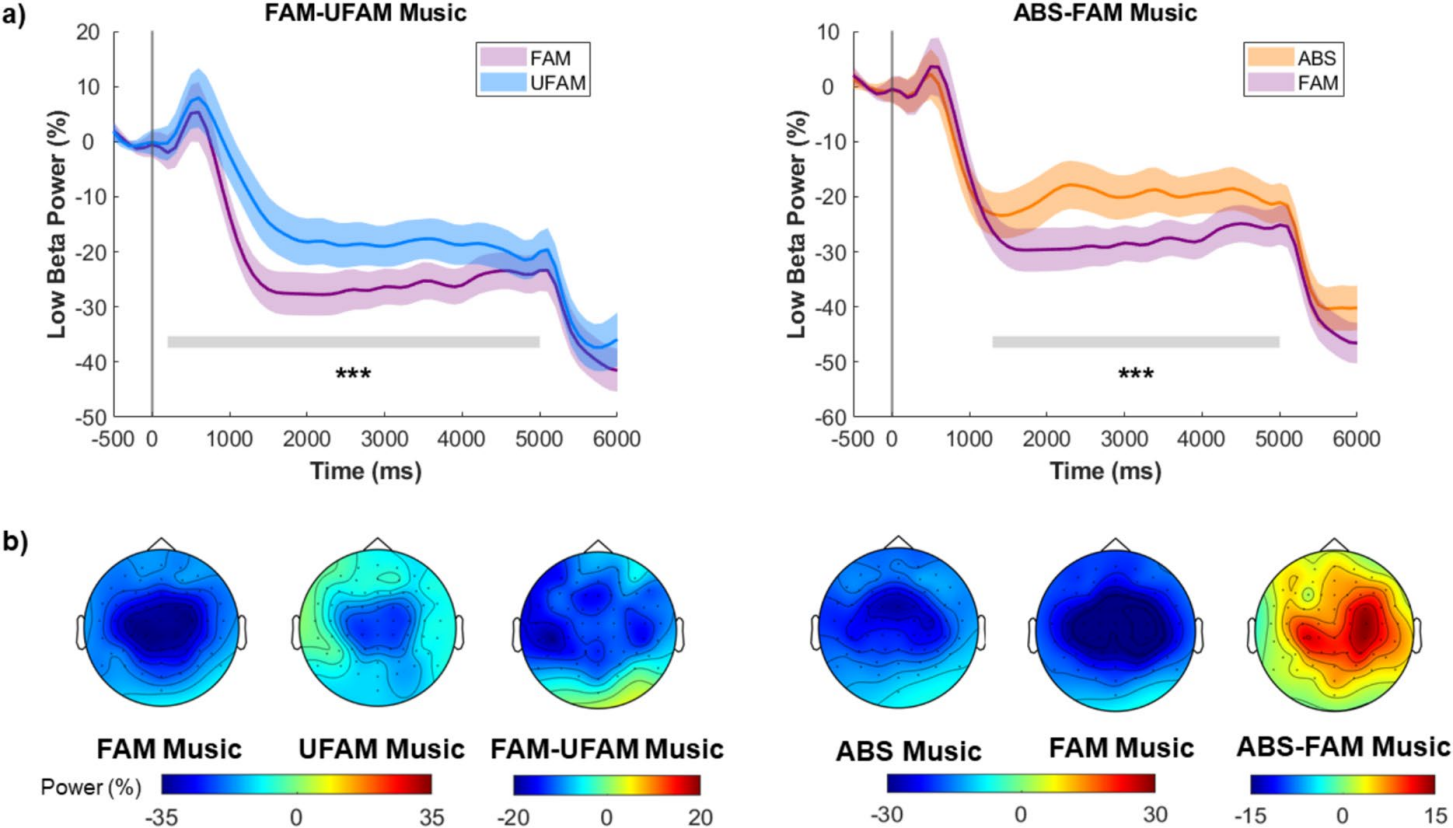
**a**) Time courses of low beta power (12 to 16 Hz) during music listening, shown relative to the pre-stimulus baseline, averaged across participants and electrodes within each significant cluster. Colored lines represent each condition (UFAM = blue, FAM = purple, ABS = orange). Shading around each line represents ±1 SEM. Time 0 indicates the onset of the stimuli. The grey bar denotes the time window of significant differences (*** *p* < 0.001). Greater low beta suppression is observed for FAM music compared to UFAM music (left panel) and ABS music (right panel). *ABS* = Autobiographically salient; *FAM* = Familiar; *UFAM* = Unfamiliar **b**) Topographic maps showing low beta power (12 to 16 Hz) distribution around the peak latency (± 300 ms). The left panel displays the distribution while listening to FAM, UFAM, and FAM-UFAM music from 1100 to 1700 ms (peak = 1400 ms); the right panel displays ABS, FAM, and ABS-FAM music from 3000 to 3600 ms (peak = 3300 ms). Power values reflect percent change from baseline, averaged across participants and displayed at each electrode. The FAM-UFAM difference map shows greater low beta suppression for FAM music, predominantly over left temporal and frontal area. The ABS-FAM difference map reveals greater suppression for FAM than ABS, with the strongest difference over the right frontal-central, central, and central-parietal regions

Figure 6a shows the temporal dynamics of low beta power activity, averaged across electrodes identified from cluster-based permutation statistics for FAM-UFAM and ABS-FAM music.

The FAM-UFAM music contrast showed greater low beta power suppression for FAM than UFAM music (*p* < 0.001). The cluster occurred from 200 to 5000 ms, with the maximum difference peaking at 1400 ms over left temporal and frontal regions (Fig. 6b, left panel).

The ABS-FAM music contrast also revealed greater low beta power suppression for FAM music relative to ABS music (*p* < 0.001). This cluster occurred between 1300 and 6000 ms, with the peak at 3300 ms, right-lateralized over the frontal-central, central, and central-parietal regions (Fig. 6b, right panel). For details on the cluster-based permutation statistics, see Table 4.

**Table 4:** Summary of Cluster-Based Permutation Statistics from Low Beta Band Power Analysis

FAM Music – UFAM Music					
Cluster	Mean FAM	Mean UFAM	*p*-value	Latency, Maximum Latency (ms)	Electrodes
1	−0.2603	−0.1534	0	200-5000, *1400*	Fp1, AF7, AF3, F1, F3, F5, F7, FT7, FC5, FC3, FC1, C1, C3, C5, T7, TP7, CP5, CP3, CP1, P1, P3, P5, P7, P9, PO7, PO3, O1, Oz, POz, Pz, CPz, Fpz, Fp2, AF8, AF4, AFz, Fz, F2, F4, F6, F8, FT8, FC6, FC4, FC2, FCz, *Cz*, C2, C4, C6, T8, TP8, CP6, CP4, CP2, P2, P4, P6, PO4

ABS Music – FAM Music					
Cluster	Mean ABS	Mean FAM	*p*-value	Latency, Maximum Latency (ms)	Electrodes
1	−0.1886	−0.2930	0	1300-5000, *3300*	Fp1, AF7, AF3, F1, F7, FC1, C1, C3, C5, TP7, CP5, CP3, CP1, P1, P3, P7, P9, PO7, PO3, O1, Oz, POz, Pz, CPz, Fpz, Fp2, AF8, AF4, AFz, Fz, F2, F4, F6, F8, FT8, FC6, FC4, FC2, FCz, Cz, C2, *C4*, C6, T8, TP8, CP6, CP4, CP2, P2, P4, P6, P8, P10, PO8, PO4, O2

*ABS* = Autobiographically salient; *FAM* = Familiar; *UFAM* = Unfamiliar; *Italicized* = Electrode at maximum amplitude

### Brain-behavior correlation results

We explored brain-behavior relationships within each music condition by correlating ERP amplitude and band-specific frequency responses (i.e., alpha and low beta power) with behavioral outcome measures, including RT, familiarity and memory ratings.

**Fig. 7:**
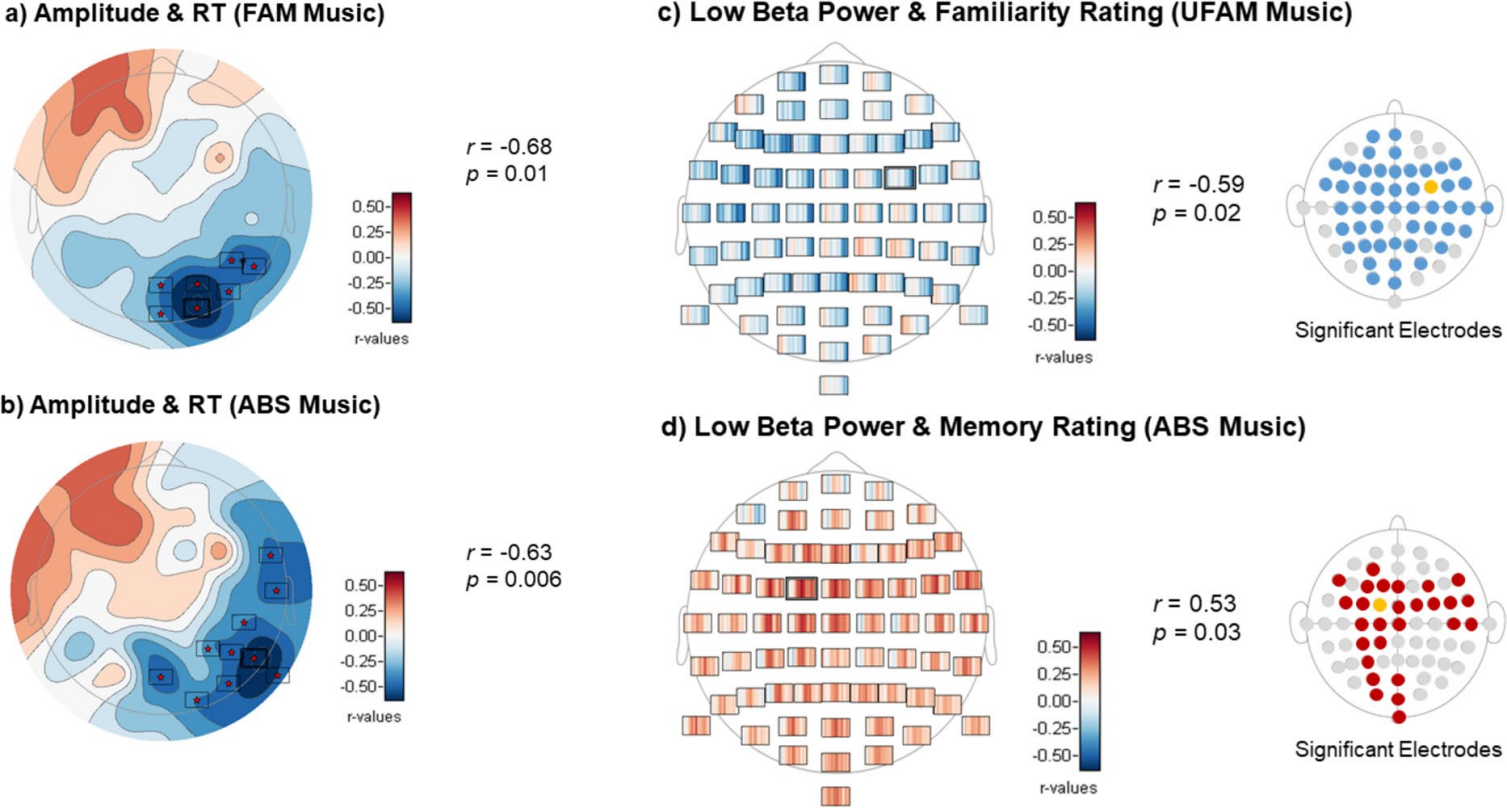
Topographic maps of significant brain-behavior correlations between **a**) ERP amplitude and RT during FAM music listening (*r* = −0.68, *p* = 0.01); **b**) ERP amplitude and RT during ABS music listening (*r* = −0.63, *p* = 0.006); **c**) Low beta power and familiarity ratings during UFAM music listening (*r =* −0.59, *p* = 0.02); and **d**) Low beta power and memory ratings during ABS music listening (*r* = 0.53, *p* = 0.03). Color maps represent Pearson *r*-values, with blue indicating negative correlations and red indicating positive correlations. For a) and b), electrodes contributing to the significant cluster identified via cluster-based permutation test are highlighted with black boxes and red stars (* *p* < 0.05). For c) and d), significant electrodes from the time-frequency clusters are displayed on the right-side montages as colored dots (blue = negative correlation, red = positive correlation, yellow = peak electrode). *ABS* = Autobiographically salient; *FAM* = Familiar; *UFAM* = Unfamiliar; *RT* = Reaction time

Cluster-based statistics revealed a moderately strong negative correlation between ERP amplitude and RT within the FAM music condition (*p* = 0.0124,* r* = −0.6873), linking higher amplitudes with faster identification of FAM excerpts. A cluster was observed in the posterior midline and right parietal-occipital regions between 477 and 5000 ms (Fig. 7a). Similarly, a significant negative correlation (*p* = 0.006, *r* = −0.6320) was also found between amplitude and RT within the ABS music condition between 488 and 5000 ms. The cluster overlapped with that identified in FAM music, including right parietal-occipital scalp areas, but extended to right temporal regions (Fig. 7b). Taken together, these associations, along with the absence of a significant brain-behavior correlation within these measures for UFAM music, suggest that larger ERP amplitudes are functionally relevant for faster recognition of FAM and ABS music.

A moderate negative association (*p* = 0.0228*, r* = −0.5930) between low beta power and familiarity ratings within the UFAM music condition was revealed between 3100 and 5000 ms, over a large, bilateral cluster that spanned frontal-central-parietal-occipital regions (Fig. 7c). The finding suggests that increased beta power is related to lower familiarity ratings. In the context of UFAM music, this may reflect the maintenance of a cognitive state in which no episodic memory is retrieved [46]. The emergence of a late cluster may also reflect increased reliance on top-down evaluative processes during decision making when exposed to unfamiliar stimuli [47].

Lastly, low beta power was positively correlated with memory ratings within the ABS music condition between 1500 and 3400 ms (*p* = 0.0344, *r* = 0.5359), with increased low beta power associated with higher memory ratings. This bilateral cluster encompassed frontal-central-parietal-occipital areas (Fig. 7d). Given the peak latency of the cluster (i.e., 2500 ms) and that identification of ABS excerpts occurred around 2100 ms (Experiment 1), the association may reflect re-experiencing of autobiographical memory representations. For a detailed list of all clusters, see Table 5.

**Table 5:** Summary of Cluster-Based Permutation Statistics from Brain-Behavior Correlation Analysis

ERP Amplitude and RT Within FAM Music				
Cluster	*p*-value	*r-value*	Latency, Maximum Latency (ms)	Electrodes
1	0.0124	−0.6873	477-5000, *1719*	Oz, POz, Pz, P2, P4, P6, P8, PO8, PO4, *O2*

ERP Amplitude and RT Within ABS Music				
Cluster	*p*-value	*r-value*	Latency, Maximum Latency (ms)	Electrodes
1	0.006	−0.6320	488-5000, *1742*	P1, P3, P5, PO3, Poz, FT8, C6, T8, CP6, CP4, CP2, P2, P4, P6, *P8*, P10, PO8, PO4, O2

Low Beta Power and Familiarity Ratings Within UFAM Music				
Cluster	*p*-value	*r-value*	Latency, Maximum Latency (ms)	Electrodes
1	0.0228	−0.5930	3100-5000, *4900*	Fp1, AF7, AF3, F1*,* F3, F5, F7, FT7, FC5, FC3, FC1, C1, C3, C5, T7, CP5, CP3, CP1, P1, P3, P5, PO3, O1, Oz, POz, Pz, CPz, Fpz, AFz, Fz, F2, F4, F6, F8, FT8, FC6, *FC4*, FC2, FCz, Cz, C2, C4, C6, T8, TP8, CP6, CP4, CP2, P2, P4, P6, P8, PO4

Low Beta Power and Memory Ratings Within ABS Music				
Cluster	*p*-value	*r-value*	Latency, Maximum Latency (ms)	Electrodes
1	0.0344	0.5359	1500-3400, *2500*	AF3, F1, F3, F7, FC5, FC3, *FC1*, C1, C3, CP3, CP1, P1, P3, PO3, O1, Iz, Oz, POz, Fz, F4, F6, F8, FT8, FC6, FC4, FC2, FCz, Cz, C2, C4, C6, T8, TP8, CP6, CP4, P2, PO4

*ABS* = Autobiographically salient; *FAM* = Familiar; *UFAM* = Unfamiliar; *ms* = Millisecond; *RT* = Reaction time; *Italicized* = Latency or electrode at maximum

## Discussion

The present study investigated older adults' behavioral and neural responses when listening to music excerpts that varied in personal significance. We were particularly interested in determining whether music associated with significant personal events, as in ABS music, generates brain responses that differ from FAM music. The first experiment showed that older adults were faster at identifying ABS music relative to FAM or UFAM music. On average, older adults took slightly over 2100 ms to correctly identify whether the incoming musical excerpt was associated with an autobiographical event. We also observed higher classification accuracy when identifying ABS than FAM and UFAM music. ERP analyses revealed differences in sustained activity with FAM and UFAM being more negative than ABS music over the right frontal-central areas. The cluster began at 2238 ms and extended until the end of the epoch, in line with the LFE. Contrastingly, neural responses associated with FAM music emerged earlier but lasted a shorter interval and displayed an LPC relative to UFAM music. Time-frequency results further supported a distinction between ABS and FAM music. Compared to the latter, ABS music exhibited less low beta power suppression. When examining brain-behavior relationships, we found significant associations between ERP amplitude and RT within FAM and ABS music conditions; low beta power and familiarity within UFAM music; and low beta power and memory within ABS music.

### Early correlates of familiarity during music listening

In the present study, we observed an increase in negativity at left frontal-central sites that began at 770 ms when listening to FAM music relative to UFAM. When using a shorter analysis epoch between 0 and 1000 ms, the topography and polarity were retained, but the cluster began earlier, at 589 ms. Our results differ from an earlier study that reported increased positivity for FAM vs. UFAM music between 400 and 450 ms [32]. This discrepancy between studies may be related to methodological differences, such as the choice of reference (i.e., average reference vs. linked mastoids), which can influence topography and amplitude. Differences in participant demographics may also play a role, as our sample included older, male and female adults, whereas the study from Malekmohammadi et al. [32] comprised of young male adults. A particularly important difference may lie in task instructions. In the present study, participants listened to musical excerpts and then made a familiarity and memory rating. However, in the Malekmohammadi et al. [32] study, participants were asked to name the title of the piece, composer, and contextual details, and then proceeded to make a familiarity judgment. Naming the title and composer of the musical excerpt may have involved semantic processing, as it requires retrieving factual information. A proposal put forth by Yovel and Paller (2004) is that the FN400 may not reflect familiarity per se, but rather, conceptual priming, which can be facilitated due to pre-existing semantic associations, particularly when tasks require participants to produce a verbal label. Further support for this interpretation is provided by Olichney et al. [48], who observed intact FN400 effects to repeated words in anterograde amnesic individuals. Since familiarity is typically disrupted in amnesia, preserving FN400 effects in these individuals suggests that the FN400 may instead index semantic priming, which is often spared.

We also observed an LPC over the left parietal scalp area, peaking at approximately 1000 ms after stimulus onset. The LPC has been strongly linked to memory processes, particularly recognition memory [26]. The LPC is larger for items that are recognized as "old" (previously seen or heard) compared to items that are "new". In the present study, the LPC was larger for ABS and FAM than for UFAM music. To our knowledge, we are the first to report an LPC for ABS and FAM music listening compared to UFAM. The ABS and FAM conditions may be analogous to old items in recognition memory tasks, while UFAM excerpts can be similar to “new” items. The latency of this memory-related activity was longer than that of prior studies using recognition memory, possibly because the recognition of musical excerpts depends on the integration of at least 200 to 500 ms [20–22]. We also observed a difference in LPC amplitude between ABS and FAM conditions, which could be related to the amount of episodic details that are attached to ABS music. While FAM music may evoke episodic memories, these are likely weaker than those elicited by ABS music.

An alternative explanation for the LPC derives from more recent work that challenges the recognition memory account, proposing instead that it reflects decision-dependent memory to guide judgment [31, 49, 50]. Ratcliffe et al. [49] used drift-diffusion model analysis to test whether the FN400 or LPC could predict drift rate, the rate at which evidence accumulates toward a decision. It was revealed that only the LPC tracked memory strength for old words. Further support comes from Brezis and colleagues [31], who modified a Remember/Know paradigm by incorporating confidence judgments before asking participants to provide a “Remember”, “Know”, or “Guess” response. They found that LPC amplitude was larger for high-confidence “Know” responses than for low-confidence “Remember” responses, undermining the notion that the LPC is exclusive to recollection. In the context of our study, this interpretation would imply that the observed effect reflects stronger memory signals that drive more confident decision-making judgments.

### Greater alpha power suppression during familiar music listening

Consistent with previous research [35], our contrast between FAM and UFAM music indicated significant power differences in the alpha band, whereby greater suppression was observed for the former condition. Alpha power suppression is a well-established marker of enhanced auditory attention [51, 52], including during music listening [35, 53, 54]. Thus, in the present study, the observed changes may reflect increased engagement and attention when listening to FAM music due to prior experience.

Relatedly, the Attention to Memory model [55] posits that the dorsal parietal cortex supports top-down, goal-directed attention, while the ventral parietal cortex mediates bottom-up, stimulus-driven processing. The former is employed during effortful retrieval. Greater alpha suppression observed for FAM relative to ABS and UFAM music suggests increased recruitment of top-down attentional processes, likely due to FAM music being recognizable yet lacking strong salience, thus prompting a memory search.

Another possible explanation for the observed event-related desynchronization of alpha power is that it may reflect predictive processes. Ito et al. [53] presented non-musicians with 20 FAM and 20 UFAM melodies, which were modified to include three seconds of silence in the middle of the excerpt. Participants were asked to predict the melody during the silent section. Time-frequency analysis revealed that relative to UFAM melodies, FAM melodies exhibited greater alpha power suppression in left frontal-central regions, just before the silent section. This suggests that the brain engages in anticipatory processing when listening to FAM music, which may also be at play in the present study.

We observed that the maximum difference of the FAM-UFAM music contrast occurred over the left frontal-temporal scalp area (FT7 electrode), broadly mapping onto the left inferior frontal gyrus. Relatedly, this brain region was one of the most commonly activated areas when listening to FAM music compared to UFAM music, which may indicate prediction processes and/or subvocalization [17]. However, given the spatial limitations of EEG and the absence of additional imaging techniques, such as fMRI, this interpretation should be taken with caution.

### Autobiographically salient music listening recruits right frontal-central regions

We hypothesized that ABS music relative to UFAM music would produce an early response indexing familiarity and subsequent difference in neural activity that may reflect a greater strength of memory association. In the present study, we observed a difference in ABS and UFAM amplitude whereby ABS generated a more positive-going modulation than UFAM music over right frontal regions from 1450 ms until the end of the epoch. This cluster is topographically similar to an early cluster found by Jagiello and colleagues [34] but differs in latency and amplitude, which can be explained by study design, including sample size, baseline and epoch length, filtering, and whether the ABS and UFAM stimuli were measurably comparable in audio attributes. It is worth noting that the next cluster observed in our study, whereby ABS music was more positive than UFAM music, beginning around 580 ms over left parietal regions, is similar to the second cluster reported by Jagiello et al. [34].

We also hypothesized an LFE amplitude difference between the ABS and FAM conditions. The contrast between ABS and FAM conditions revealed a difference in sustained activity over right frontal-central regions. Three propositions have been put forth concerning the function of the LFE. One suggestion is that it indexes post-retrieval processing [27, 28, 56]. The second is that the LFE indexes the retrieval of self-relevant information [29, 30]. Lastly, the LFE has been associated with retrieval effort when memory representations are weak [57]. The latter seems unlikely given that participants provided specific song titles and artists'names, which prompt a memory of a significant person, place, or event. Further, ABS music is deeply encoded, possesses a unique quality of involuntary retrieval and is emotionally rich. Therefore, our results align with post-retrieval processing and/or self-relevant representations.

###  Less beta power suppression during autobiographically salient music listening

Our time-frequency analysis revealed that the ABS-FAM music contrast was primarily marked by changes in the low beta band. Specifically, FAM music elicited greater suppression than ABS music, peaking over the midline-right central scalp area (C2 electrode). Given that motor imagery can lead to event-related desynchronization over sensorimotor areas [58, 59], this may suggest that FAM music evoked stronger, more automatic motor tendencies, such as an urge to tap or move along to the music, which participants actively suppressed during the task. The effect was observed over the right central scalp area, a site corresponding to sensorimotor areas including the supplementary motor cortex, which has been linked to FAM music listening [17]. A contributing factor to the observed effect is the frequency of exposure to FAM compared to ABS music. While ABS music was selected by participants based on its significance and associations with important people, places, and events, it does not necessarily follow that these songs were frequently listened to in daily life. In contrast, FAM music consisted of widely popular songs that were often listed as a “hit” on the Billboard charts (see Table 1, Supplementary Material), and thus, frequently played on the radio or featured in films and other media. This increased exposure may have reinforced sensorimotor associations over time, leading to more tightly linked auditory-motor responses.

### Musical memory: Single- or dual-process model?

Two theoretical accounts have been proposed in recognition memory. The single-process model posits that recognition judgments are based on the strength of an item’s memory signal, which reflects the degree to which the stimulus is similar to stored information [60]. Neural responses scale with memory strength, such that low recollection falls below a decision threshold, leading to weaker neural responses, and high recollection exceeds the threshold, producing stronger neural responses. This model therefore predicts a graded effect, with the ERP amplitude indexing the strength of the memory trace [27, 61]. Contrastingly, the dual-process model posits temporally and topographically distinct modulations associated with familiarity and recollection [62]. According to this account, recognition memory involves an early sense of familiarity, as indexed by the FN400, followed by a later recollection response, marked by the LPC (for a review, see Yonelinas [63]). Lastly, although the late right frontal positivity effect has not been tied to any particular model, it is often associated with source memory decisions. Indeed, damage to the prefrontal cortex has been associated with impaired source memory performance [64, 65].

Our findings further support the dual process account by revealing distinct neurophysiological activity when listening to music of varying levels of personal significance. The observed enhanced positivity for ABS and FAM conditions over the left parietal area may index familiarity, which is larger in amplitude for ABS than FAM music. More importantly, we observed a difference in sustained activity over the right frontal-central region throughout the musical excerpt, characterized by larger negativity for FAM than ABS music. The timing, amplitude distribution, and specificity of the neural responses indicate distinct memory-related processes that support music-evoked recollection.

Although our study bears some conceptual resemblance to the study-test paradigms often employed in recognition memory, the present design diverges in certain aspects. First, there were no additions of “new” stimuli, making it difficult to draw direct inferences between “old” and “new” material. Second, confidence ratings are traditionally included in study-test paradigms to further distinguish between familiarity-based and recollection-based recognition, which were not collected in the present study. Future studies could incorporate confidence ratings to determine whether the LPC observed in FAM music listening is related to decision making. Third, study-test paradigms do not typically include stimuli that are deeply relevant to the individual. Thus, the element of autobiographical memory and associated emotions may introduce a layer of complexity. Relatedly, the use of music, which evolves over time, may evoke neural responses that differ from those that arise from more static stimuli, such as words and images. Despite these differences in task design, we found strong evidence for distinct scalp distributions and latencies between FAM and ABS music, such as the LPC and the LFE, which point towards a model that involves more than one process in the context of musical memory.

### Limitations

We sought to investigate whether and how music that engages autobiographical memory processes is distinct from music elicited by familiarity-based processing by comparing neural activity while listening to ABS, FAM and UFAM music. Using a RT task, we first established the time required for older adults to correctly identify music from each condition. Although we used 15 songs per condition and randomized the presentation of the clips to reduce the likelihood of a habitual response, one limitation was that each excerpt was repeated multiple times and consequently may have become more familiar over the course of the experiment. Future studies could use excerpts clipped from various parts of the same song while maintaining a large set of stimuli to minimize repeated exposure.

Music is a complex stimulus due to its constituent features (i.e., rhythm, timbre, etc.), and the dynamic nature of one or more musical elements could influence the observed neural responses. Across conditions, our acoustic analysis of the stimuli showed no significant differences in root mean square intensity, tempo, or zero crossing rate between musical excerpts from the three conditions. However, there were significant differences in onset rise time, pitch, spectral centroid, and spectral bandwidth. Nevertheless, low-level acoustic differences would be reflected in the early portion of the evoked potential (i.e., N1, P2) and are, therefore, unlikely to have affected long latency responses.

Finally, we did not collect emotion ratings during the task. As a result, we cannot directly assess the extent to which emotional aspects of autobiographical remembering contributed to the observed neural responses. Notably, the ERPs for the ABS music were right-lateralized in frontal-central regions, a pattern that aligns with previous neuroimaging studies linking right-hemisphere activity to emotional aspects of autobiographical memory retrieval [66]. Further, given that our sample included older adults, in some cases, their ABS songs included music associated with loved ones who have passed away, suggesting that the stimuli may have evoked emotionally charged memories. However, without subjective measures of emotion, this interpretation remains speculative. More EEG research is needed to elucidate the timing and scalp distribution of emotional processes when listening to ABS songs, including distinctions among emotions such as happiness, sadness, and more complex emotions, such as nostalgia.

## Conclusion

Our behavioral and electrophysiological data provide converging evidence that ABS music uniquely engages memory-related processes. Specifically, ABS music elicits quicker RTs and distinct neural signatures than FAM music. The difference in ERPs between these conditions was localized over the right frontal-central scalp area whereby FAM music displayed greater sustained negativity compared to ABS music. In contrast, listening to FAM music was associated with greater positivity than UFAM music over the left posterior-occipital regions, similar to an LPC. Time-frequency analyses revealed less low beta suppression for ABS music than FAM music, while conversely, FAM music showed greater alpha suppression than UFAM music. Brain-behavior correlations further highlight negative relationships between ERP amplitude and RT within FAM and ABS music conditions. Low-beta power was negatively correlated with familiarity ratings within the UFAM condition and positively correlated with memory ratings within the ABS condition. Taken together, the temporal, spectral, and topographic differences between FAM and ABS music add to the evidence that distinct cognitive and neural processes underpin familiarity and deeper recollection. These findings underscore the need to distinguish between the two conditions in musical memory studies and offer meaningful implications for ABS music-based interventions in aging and memory-related disorders, such as dementia.

## Supplementary Information

Below is the link to the electronic supplementary material.

Supplementary file1 (DOCX 34.9 KB)

## Data Availability

The data that support findings of this study are available upon request from the corresponding author.

## References

[1] Addis DR, Musicaro R, Pan L, Schacter DL. Episodic simulation of past and future events in older adults: Evidence from an experimental recombination task. Psychol Aging. 2010;25:369–76. 10.1037/a0017280. 20545421 10.1037/a0017280 PMC2896213

[2] Frankenberg C, Knebel M, Degen C, Siebert JS, Wahl H-W, Schröder J. Autobiographical memory in healthy aging: A decade-long longitudinal study. Aging Neuropsychol Cogn. 2022;29:158–79. 10.1080/13825585.2020.1859082. 10.1080/13825585.2020.1859082 33402012

[3] Piolino P, Coste C, Martinelli P, Macé A-L, Quinette P, Guillery-Girard B, et al. Reduced specificity of autobiographical memory and aging: Do the executive and feature binding functions of working memory have a role? Neuropsychologia. 2010;48:429–40. 10.1016/j.neuropsychologia.2009.09.035. 19804792 10.1016/j.neuropsychologia.2009.09.035

[4] Piolino P, Desgranges B, Clarys D, Guillery-Girard B, Taconnat L, Isingrini M, et al. Autobiographical memory, autonoetic consciousness, and self-perspective in aging. Psychol Aging. 2006;21:510–25. 10.1037/0882-7974.21.3.510. 16953713 10.1037/0882-7974.21.3.510

[5] Conway MA, Pleydell-Pearce CW. The construction of autobiographical memories in the self-memory system. Psychol Rev. 2000;107:261–88. 10.1037/0033-295X.107.2.261. 10789197 10.1037/0033-295x.107.2.261

[6] Tulving E. Elements of episodic memory. Oxford: Clarendon; 1983.

[7] Rathbone CJ, O’Connor AR, Moulin CJA. The tracks of my years: Personal significance contributes to the reminiscence bump. Mem Cognit. 2017;45:137–50. 10.3758/s13421-016-0647-2. 27566486 10.3758/s13421-016-0647-2

[8] Zator K, Katz AN. The language used in describing autobiographical memories prompted by life period visually presented verbal cues, event-specific visually presented verbal cues and short musical clips of popular music. Memory. 2017;25:831–44. 10.1080/09658211.2016.1224353. 27580165 10.1080/09658211.2016.1224353

[9] Belfi AM, Karlan B, Tranel D. Music evokes vivid autobiographical memories. Memory. 2016;24:979–89. 10.1080/09658211.2015.1061012. 26259098 10.1080/09658211.2015.1061012

[10] Greasley AE, Lamont A. Exploring engagement with music in everyday life using experience sampling methodology. Music Sci. 2011;15:45–71. 10.1177/1029864910393417.

[11] Schulkind MD, Hennis LK, Rubin DC. Music, emotion, and autobiographical memory: they’re playing your song. Mem Cognit. 1999;27:948–55. 10.3758/bf03201225. 10586571 10.3758/bf03201225

[12] Merriam AP. The anthropology of music. Evanston, IL: Northwestern Univ. Press; 2006.

[13] Cuddy LL. Long-Term Memory for Music. In: Bader R, editor. Springer Handb. Syst. Musicol., Berlin, Heidelberg: Springer Berlin Heidelberg; 2018;453–9. 10.1007/978-3-662-55004-5_23.

[14] El Haj M, Fasotti L, Allain P. The involuntary nature of music-evoked autobiographical memories in Alzheimer’s disease. Conscious Cogn. 2012;21:238–46. 10.1016/j.concog.2011.12.005. 22265372 10.1016/j.concog.2011.12.005

[15] El Haj M, Antoine P, Nandrino JL, Gély-Nargeot M-C, Raffard S, Brodaty H. Self-defining memories during exposure to music in Alzheimer’s disease. Int Psychogeriatr. 2015;27:1719–30. 10.1017/S1041610215000812. 26018841 10.1017/S1041610215000812

[16] Fischer CE, Churchill N, Leggieri M, Vuong V, Tau M, Fornazzari LR, et al. Long-known music exposure effects on brain imaging and cognition in early-stage cognitive decline: A pilot study. J Alzheimers Dis. 2021;84:819–33. 10.3233/JAD-210610. 34602475 10.3233/JAD-210610

[17] Vuong V, Hewan P, Perron M, Thaut MH, Alain C. The neural bases of familiar music listening in healthy individuals: An activation likelihood estimation meta-analysis. Neurosci Biobehav Rev. 2023;154:105423. 10.1016/j.neubiorev.2023.105423. 37839672 10.1016/j.neubiorev.2023.105423

[18] Thaut MH, Fischer CE, Leggieri M, Vuong V, Churchill NW, Fornazzari LR, et al. Neural basis of long-term musical memory in cognitively impaired older persons. Alzheimer Dis Assoc Disord. 2020;34:267–71. 10.1097/WAD.0000000000000382. 32384286 10.1097/WAD.0000000000000382

[19] Bella SD, Peretz I, Aronoff N. Time course of melody recognition: A gating paradigm study. Percept Psychophys. 2003;65:1019–28. 10.3758/BF03194831. 14674630 10.3758/bf03194831

[20] Filipic S, Tillmann B, Bigand E. Judging familiarity and emotion from very brief musical excerpts. Psychon Bull Rev. 2010;17:335–41. 10.3758/PBR.17.3.335. 20551355 10.3758/PBR.17.3.335

[21] Tillmann B, Albouy P, Caclin A, Bigand E. Musical familiarity in congenital amusia: Evidence from a gating paradigm. Cortex. 2014;59:84–94. 10.1016/j.cortex.2014.07.012. 25151640 10.1016/j.cortex.2014.07.012

[22] Schellenberg EG, Iverson P, Mckinnon MC. Name that tune: Identifying popular recordings from brief excerpts. Psychon Bull Rev. 1999;6:641–6. 10.3758/BF03212973. 10682207 10.3758/bf03212973

[23] Krumhansl CL. Plink: “Thin slices” of music. Music Percept. 2010;27:337–54. 10.1525/mp.2010.27.5.337.

[24] Rugg MD, Curran T. Event-related potentials and recognition memory. Trends Cogn Sci. 2007;11:251–7. 10.1016/j.tics.2007.04.004. 17481940 10.1016/j.tics.2007.04.004

[25] Friedman D, Johnson R. Event-related potential (ERP) studies of memory encoding and retrieval: a selective review. Microsc Res Tech. 2000;51:6–28. 10.1002/1097-0029(20001001)51:1<6::AID-JEMT2>3.0.CO;2-R. 11002349 10.1002/1097-0029(20001001)51:1<6::AID-JEMT2>3.0.CO;2-R

[26] Duarte A, Ranganath C, Winward L, Hayward D, Knight RT. Dissociable neural correlates for familiarity and recollection during the encoding and retrieval of pictures. Cogn Brain Res. 2004;18:255–72. 10.1016/j.cogbrainres.2003.10.010. 10.1016/j.cogbrainres.2003.10.010 14741312

[27] Wilding EL, Rugg MD. An event-related potential study of recognition memory with and without retrieval of source. Brain. 1996;119:889–905. 10.1093/brain/119.3.889. 8673500 10.1093/brain/119.3.889

[28] Wilding EL. Separating retrieval strategies from retrieval success: an event-related potential study of source memory. Neuropsychologia. 1999;37:441–54. 10.1016/S0028-3932(98)00100-6. 10215091 10.1016/s0028-3932(98)00100-6

[29] Kotlewska I, Nowicka A. Present-self, past-self and the close-other: neural correlates of assigning trait adjectives to oneself and others. Eur J Neurosci. 2016;44:2064–71. 10.1111/ejn.13293. 27285486 10.1111/ejn.13293

[30] Magno E, Allan K. Self-reference during explicit memory retrieval: An event-related potential analysis. Psychol Sci. 2007;18:672–7. 10.1111/j.1467-9280.2007.01957.x. 17680935 10.1111/j.1467-9280.2007.01957.x

[31] Brezis N, Bronfman ZZ, Yovel G, Goshen-Gottstein Y. The electrophysiological signature of remember-know is confounded with memory strength and cannot be interpreted as evidence for dual-process theory of recognition. J Cogn Neurosci. 2017;29:322–36. 10.1162/jocn_a_01053. 27991029 10.1162/jocn_a_01053

[32] MacKenzie G, Donaldson DI. Dissociating recollection from familiarity: Electrophysiological evidence that familiarity for faces is associated with a posterior old/new effect. NeuroImage. 2007;36:454–63. 10.1016/j.neuroimage.2006.12.005. 17451972 10.1016/j.neuroimage.2006.12.005

[33] Yovel G, Paller KA. The neural basis of the butcher-on-the-bus phenomenon: when a face seems familiar but is not remembered. NeuroImage. 2004;21:789–800. 10.1016/j.neuroimage.2003.09.034. 14980582 10.1016/j.neuroimage.2003.09.034

[34] Jagiello R, Pomper U, Yoneya M, Zhao S, Chait M. Rapid brain responses to familiar vs. unfamiliar music - an EEG and pupillometry study. Sci Rep 2019;9:15570. 10.1038/s41598-019-51759-9. 10.1038/s41598-019-51759-9 PMC6821741 31666553

[35] Malekmohammadi A, Ehrlich SK, Rauschecker JP, Cheng G. Listening to familiar music induces continuous inhibition of alpha and low-beta power. J Neurophysiol. 2023;129:1344–58. 10.1152/jn.00269.2022. 37141051 10.1152/jn.00269.2022

[36] Chow R, Rabi R, Paracha S, Hasher L, Anderson ND, Alain C. Default mode network and neural phase synchronization in healthy aging: A resting state EEG study. Neuroscience. 2022;485:116–28. 10.1016/j.neuroscience.2022.01.008. 35051530 10.1016/j.neuroscience.2022.01.008

[37] Grady CL, Garrett DD. Brain signal variability is modulated as a function of internal and external demand in younger and older adults. NeuroImage. 2018;169:510–23. 10.1016/j.neuroimage.2017.12.031. 29253658 10.1016/j.neuroimage.2017.12.031

[38] Recording Industry Association of America. RIAA gold & platnium program [Internet]. RIAA 2024. https://www.riaa.com/gold-platinum/about-awards/. Accessed 19 Jul 2024.

[39] Audacity Team. Audacity(R): Free audio editor and recorder. [Computer program]. Muse Group; 2023. https://www.audacityteam.org/. Accessed 4 Jan 2023.

[40] Boersma P, Weenink D. Praat: doing phonetics by computer. [Computer program]. 2024. https://praat.org. Accessed 3 Jan 2023.

[41] Neurobehavioral Systems, Inc. Presentation(R). [Computer program]. 2025. https://www.neurobs.com. Accessed 19 Dec 2022.

[42] R Core Team. R: A Language and Environment for Statistical Computing. Vienna, Austria: R Foundation for Statistical Computing; 2025.

[43] Maris E, Oostenveld R. Nonparametric statistical testing of EEG- and MEG-data. J Neurosci Methods. 2007;164:177–90. 10.1016/j.jneumeth.2007.03.024. 17517438 10.1016/j.jneumeth.2007.03.024

[44] Lütkenhöner B, Olaf Steinsträter. High-precision neuromagnetic study of the functional organization of the human auditory cortex. Audiol Neurotol 1998;3:191–213. 10.1159/000013790. 10.1159/000013790 9575385

[45] Picton TW, Alain C, Woods DL, John MS, Scherg M, Valdes-Sosa P, et al. Intracerebral sources of human auditory-evoked potentials. Audiol Neurotol. 1999;4:64–79. 10.1159/000013823. 10.1159/000013823 9892757

[46] Engel AK, Fries P. Beta-band oscillations — signalling the status quo? Curr Opin Neurobiol. 2010;20:156–65. 10.1016/j.conb.2010.02.015. 20359884 10.1016/j.conb.2010.02.015

[47] Haegens S, Nácher V, Hernández A, Luna R, Jensen O, Romo R. Beta oscillations in the monkey sensorimotor network reflect somatosensory decision making. Proc Natl Acad Sci. 2011;108:10708–13. 10.1073/pnas.1107297108. 21670296 10.1073/pnas.1107297108 PMC3127887

[48] Olichney JM. Word repetition in amnesia: Electrophysiological measures of impaired and spared memory. Brain. 2000;123:1948–63. 10.1093/brain/123.9.1948. 10960058 10.1093/brain/123.9.1948

[49] Ratcliff R, Sederberg PB, Smith TA, Childers R. A single trial analysis of EEG in recognition memory: Tracking the neural correlates of memory strength. Neuropsychologia. 2016;93:128–41. 10.1016/j.neuropsychologia.2016.09.026. 27693702 10.1016/j.neuropsychologia.2016.09.026 PMC5148728

[50] Yang H, Laforge G, Stojanoski B, Nichols ES, McRae K, Köhler S. Late positive complex in event-related potentials tracks memory signals when they are decision relevant. Sci Rep. 2019;9:9469. 10.1038/s41598-019-45880-y. 31263156 10.1038/s41598-019-45880-y PMC6603184

[51] Teoh ES, Lalor EC. EEG decoding of the target speaker in a cocktail party scenario: considerations regarding dynamic switching of talker location. J Neural Eng. 2019;16: 036017. 10.1088/1741-2552/ab0cf1. 30836345 10.1088/1741-2552/ab0cf1

[52] Shen D, Ross B, Alain C. Temporal cuing modulates alpha oscillations during auditory attentional blink. Eur J Neurosci. 2016;44:1833–45. 10.1111/ejn.13266. 27152668 10.1111/ejn.13266

[53] Ito S, Matsunaga K, Chanpornpakdi I, Tanaka T. Effect of predicting familiar melodies on alpha power. 2023. 10.1101/2023.03.16.532954.

[54] Jäncke L, Leipold S, Burkhard A. The neural underpinnings of music listening under different attention conditions. NeuroReport. 2018;29:594–604. 10.1097/WNR.0000000000001019. 29596153 10.1097/WNR.0000000000001019

[55] Cabeza R, Mazuz YS, Stokes J, Kragel JE, Woldorff MG, Ciaramelli E, et al. Overlapping Parietal Activity in Memory and Perception: Evidence for the Attention to Memory Model. J Cogn Neurosci. 2011;23:3209–17. 10.1162/jocn_a_00065. 21568633 10.1162/jocn_a_00065 PMC3518433

[56] Rugg MD, Otten LJ, Henson RNA. The neural basis of episodic memory: evidence from functional neuroimaging. Philos Trans R Soc Lond B Biol Sci. 2002;357:1097–110. 10.1098/rstb.2002.1102. 12217177 10.1098/rstb.2002.1102 PMC1693015

[57] Henson RNA, Rugg MD, Shallice T, Dolan RJ. Confidence in recognition memory for words: Dissociating right prefrontal roles in episodic retrieval. J Cogn Neurosci. 2000;12:913–23. 10.1162/08989290051137468. 11177413 10.1162/08989290051137468

[58] Neuper C, Schlögl A, Pfurtscheller G. Enhancement of left-right sensorimotor EEG differences during feedback-regulated motor imagery. J Clin Neurophysiol. 1999;16:373–82. 10.1097/00004691-199907000-00010. 10478710 10.1097/00004691-199907000-00010

[59] Pfurtscheller G, Lopes Da Silva FH. Event-related EEG/MEG synchronization and desynchronization: basic principles. Clin Neurophysiol 1999;110:1842–57. 10.1016/S1388-2457(99)00141-8. 10.1016/s1388-2457(99)00141-8 10576479

[60] Dennis S, Humphreys MS. A context noise model of episodic word recognition. Psychol Rev. 2001;108:452–78. 10.1037/0033-295X.108.2.452. 11381837 10.1037/0033-295x.108.2.452

[61] Rugg MD, Cox CJC, Doyle MC, Wells T. Event-related potentials and the recollection of low and high frequency words. Neuropsychologia. 1995;33:471–84. 10.1016/0028-3932(94)00132-9. 7617156 10.1016/0028-3932(94)00132-9

[62] Curran T. Brain potentials of recollection and familiarity. Mem Cognit. 2000;28:923–38. 10.3758/BF03209340. 11105518 10.3758/bf03209340

[63] Yonelinas AP. The nature of recollection and familiarity: A review of 30 years of research. J Mem Lang. 2002;46:441–517. 10.1006/jmla.2002.2864.

[64] Janowsky JS, Shimamura AP, Squire LR. Source memory impairment in patients with frontal lobe lesions. Neuropsychologia. 1989;27:1043–56. 10.1016/0028-3932(89)90184-X. 2797412 10.1016/0028-3932(89)90184-x

[65] Johnson MK, Kounios J, Nolde SF. Electrophysiological brain activity and memory source monitoring. NeuroReport. 1997;8:1317–20. 10.1097/00001756-199703240-00051. 9175136 10.1097/00001756-199703240-00051

[66] Fink GR, Markowitsch HJ, Reinkemeier M, Bruckbauer T, Kessler J, Heiss W-D. Cerebral representation of one’s own past: Neural networks involved in autobiographical memory. J Neurosci. 1996;16:4275–82. 10.1523/JNEUROSCI.16-13-04275.1996. 8753888 10.1523/JNEUROSCI.16-13-04275.1996 PMC6579004

